# Letter from Quincy, Ill.

**Published:** 1883-09

**Authors:** C. B. Ellis

**Affiliations:** Quincy, Ills.


### ARTICLE XIV.

QUINCY, ILLS., July 9, 1883.

D. R. BROWER, M.D., Chicago.

*Dear Doctor:*—As I administered the anæsthetic mixture for Dr. W. A. Byrd in the two cases in which the "untoward results" supervened, I will explain how it came about. In both instances the operation was begun with a quantity of the mixture which proved to be insufficient, and the attempt was made in the first case to keep up the anæsthetic influence with chloroform. As soon as the patient inhaled the chloroform, the pallor and interruption of the heart's action became suddenly so pronounced that the operation had to be continued without any further anæsthetic influence. This case was a castration on account of tubercular deposit, and the bad symptoms were not from shock, as the cord was not yet severed.

In the second case, which was an operation for a lacerated cervix, ether was used when the mixture gave out, and as soon as it was breathed carbonic narcosis was quickly observed and the inhaler was removed, and no further influence kept up.

In all other cases where I have administered the mixture, nothing unfavorable or unpleasant occurred, except the cases where vomiting occurred, as spoken of by Dr. B. The patients are readily brought under the influence of the mixture, and are as readily and pleasantly freed from it.

I would say that pure drugs have always been used in its preparation. Hoping that others may have as pleasant experience with the anæsthetic mixture, I am,

Yours respectfully, C. B. ELLIS.

---

IN reply to a letter from the editor of the *Medical News*, Philadelphia), Dr. J. S. Billings, in charge of the library of the Surgeon-General's office, states that books are loaned from the library to other libraries which undertake to be responsible for them and have suitable buildings for their safe preservation.

Books which can readily be replaced, if lost, are also loaned to individuals upon their making a deposit with the librarian, of funds sufficient to make good any damage or loss.

Books must be sent by express, not by mail, and the cost of expressage paid by the borrower, the funds to be returned when the books are returned in good condition.

